# First person – Talia Nasr

**DOI:** 10.1242/dmm.048899

**Published:** 2021-02-04

## Abstract

First Person is a series of interviews with the first authors of a selection of papers published in Disease Models & Mechanisms, helping early-career researchers promote themselves alongside their papers. Talia Nasr is first author on ‘Disruption of a Hedgehog-Foxf1-Rspo2 signaling axis leads to tracheomalacia and a loss of Sox9^+^ tracheal chondrocytes’, published in DMM. Talia is a MD/PhD student at the University of Cincinnati, USA, currently in the final two MD years, with the PhD work completed in the lab of Aaron Zorn, investigating the pathogenesis of congenital tracheoesophageal defects.


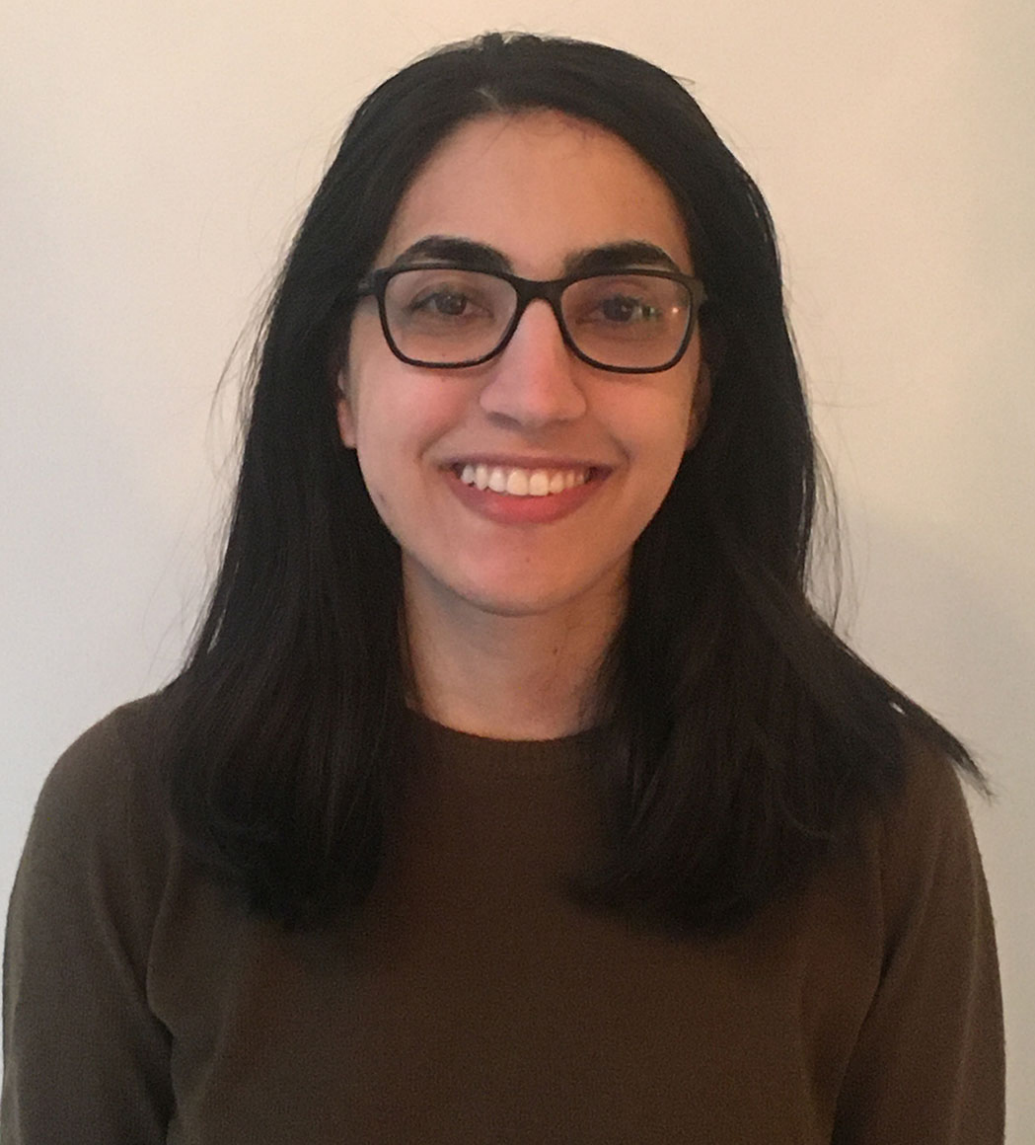


**Talia Nasr**

**How would you explain the main findings of your paper to non-scientific family and friends?**

Development of tracheal cartilage, which helps maintain an open airway, requires the protein Sox9. Our paper finds a time-specific role for the Hedgehog signalling pathway and the protein Foxf1, a Hedgehog target, to support Sox9 in the trachea. This work also reveals a potential signalling mechanism for tracheal cartilage development that involves Hedgehog, Foxf1, the Wnt signalling pathway and Sox9. Ultimately, these results show potential ways in which congenital defects in human tracheal chondrogenesis might develop.

**What are the potential implications of these results for your field of research?**

These results suggest that Hedgehog and Wnt signalling in the trachea are fairly intertwined, and warrant a deeper dive into how these two pathways interact, considering that they're both required for tracheal development. These results also lead to more questions about the specific targets of Foxf1 during tracheal development. They really make me wonder whether Foxf1 might directly bind to some unexpected genes that have been previously implicated in human congenital tracheal defects.

**What are the main advantages and drawbacks of the model system you have used as it relates to the disease you are investigating?**

Mouse tracheal patterning appears to be quite similar to that of humans, so mice can be used to model human tracheal cartilage defects. Mouse genetics, though, can be complicated and require significant time to properly set up and support.

**What has surprised you the most while conducting your research?**

I was continually amazed at the time-specific roles for Hedgehog and Foxf1 that our results showed. For example, early Cre-mediated deletion of Foxf1 led to a single foregut tube, whereas Cre-mediated deletion starting just about a day later didn't seem to have any significant impact on tracheoesophageal separation at all. An embryo's integration of so many moving parts during development, especially in such a small timeframe, is always so impressive to me.

“An embryo's integration of so many moving parts during development, especially in such a small timeframe, is always so impressive to me.”

**Describe what you think is the most significant challenge impacting your research at this time and how will this be addressed over the next 10 years?**

COVID-19 has really significantly disrupted how everyone does research, and I think we're all going to be continually figuring out what is our ‘new normal’ for doing science, and life in general, for a long time to come.

**Figure:**
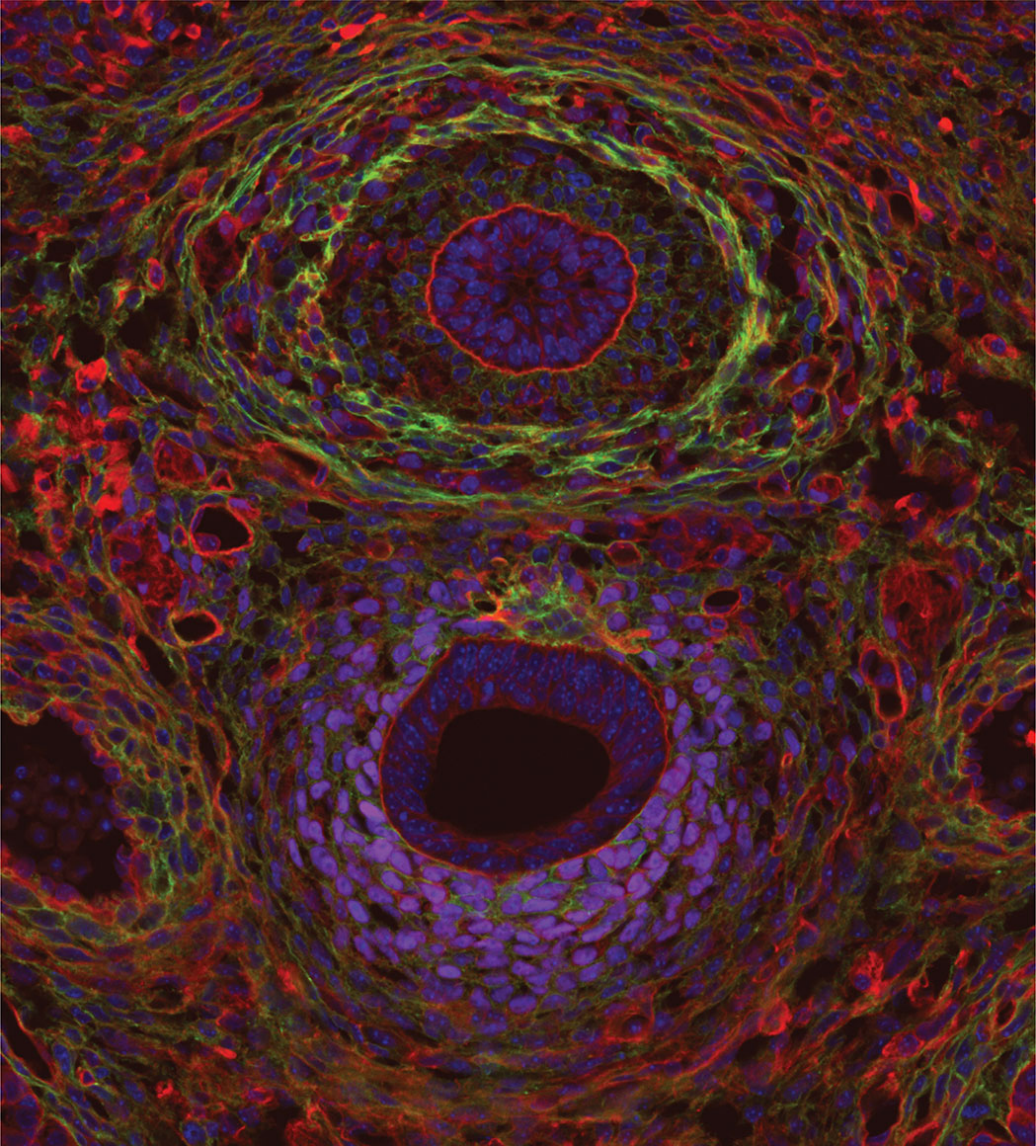
**A developing mouse oesophagus and trachea during differentiation of smooth muscle and cartilage lineages.**

**What changes do you think could improve the professional lives of early-career scientists?**

I think continuing discussions and implementing changes to foster, recruit and support a diverse academia is a good place to start. I also always find it helpful to learn more about the actual business of running a lab, as I don't think that gets openly discussed enough with trainees who are considering careers in academia.

**What's next for you?**

I'm currently completing the MD portion of my combined MD/PhD degree with the goal of eventually entering a paediatric residency program that integrates research with clinical training.
